# Volumetric accuracy of different imaging modalities in acute intracerebral hemorrhage

**DOI:** 10.1186/s12880-022-00735-3

**Published:** 2022-01-15

**Authors:** Frieder Schlunk, Johannes Kuthe, Peter Harmel, Heinrich Audebert, Uta Hanning, Georg Bohner, Michael Scheel, Justus Kleine, Jawed Nawabi

**Affiliations:** 1 Department of Neuroradiology, Charité – Universitätsmedizin Berlin, Corporate Member of Freie Universität Berlin, Humboldt-Universität Zu Berlin, and Berlin Institute of Health, Charitéplatz 1, 10117 Berlin, Germany; 2 Berlin Institute of Health (BIH), BIH Biomedical Innovation Academy, Berlin, Germany; 3 Department of Neurology, Charité - Universitätsmedizin Berlin, Campus Mitte, Humboldt-Universität Zu Berlin, Freie Universität Berlin, Charitéplatz 1, 10117 Berlin, Germany; 4 Department of Diagnostic and Interventional Neuroradiology, University Medical Center Hamburg Eppendorf, Hamburg, Germany; 5 Department of Radiology (CCM), Charité - Universitätsmedizin Berlin, Campus Mitte, Humboldt-Universität Zu Berlin, Freie Universität Berlin, Charitéplatz 1, 10117 Berlin, Germany

**Keywords:** Acute stroke, Imaging, Intracerebral hemorrhage, Neurocritical care, Neuroradiology

## Abstract

**Background:**

Follow-up imaging in intracerebral hemorrhage is not standardized and radiologists rely on different imaging modalities to determine hematoma growth. This study assesses the volumetric accuracy of different imaging modalities (MRI, CT angiography, postcontrast CT) to measure hematoma size.

**Methods:**

28 patients with acute spontaneous intracerebral hemorrhage referred to a tertiary stroke center were retrospectively included between 2018 and 2019. Inclusion criteria were (1) spontaneous intracerebral hemorrhage (supra- or infratentorial), (2) noncontrast CT imaging performed on admission, (3) follow-up imaging (CT angiography, postcontrast CT, MRI), and (4) absence of hematoma expansion confirmed by a third cranial image within 6 days. Two independent raters manually measured hematoma volume by drawing a region of interest on axial slices of admission noncontrast CT scans as well as on follow-up imaging (CT angiography, postcontrast CT, MRI) using a semi-automated segmentation tool (Visage image viewer; version 7.1.10). Results were compared using Bland–Altman plots.

**Results:**

Mean admission hematoma volume was 18.79 ± 19.86 cc. All interrater and intrarater intraclass correlation coefficients were excellent (1; IQR 0.98–1.00). In comparison to hematoma volume on admission noncontrast CT volumetric measurements were most accurate in patients who received postcontrast CT (bias of − 2.47%, SD 4.67: n = 10), while CT angiography often underestimated hemorrhage volumes (bias of 31.91%, SD 45.54; n = 20). In MRI sequences intracerebral hemorrhage volumes were overestimated in T2* (bias of − 64.37%, SD 21.65; n = 10). FLAIR (bias of 6.05%, SD 35.45; n = 13) and DWI (bias of-14.6%, SD 31.93; n = 12) over- and underestimated hemorrhagic volumes.

**Conclusions:**

Volumetric measurements were most accurate in postcontrast CT while CT angiography and MRI sequences often substantially over- or underestimated hemorrhage volumes.

**Supplementary Information:**

The online version contains supplementary material available at 10.1186/s12880-022-00735-3.

## Background

Intracerebral hemorrhage (ICH) is a deadly subtype of stroke and improvement in clinical management is urgently needed. Approximately one third of patients suffer from hematoma expansion after hospital arrival constituting a target for treatment and warranting prompt diagnosis [1]. In the acute setting, non-contrast computed tomography (NCCT) is widely used for initial diagnosis, however, time point and modality of follow-up imaging are typically not standardized [2]. In the clinical course often contrast-enhanced CT scans such as CT-angiography (CTA) or postcontrast CT and MRI are performed to evaluate a secondary underlying cause for ICH, such as cerebral amyloid angiopathy (CAA), neoplastic disease, vasculitis, cavernoma, or other vascular abnormalities [3]. Thus, neuroradiologists rely on a different imaging modality for hematoma measurement with intermodal comparison to NCCT admission imaging [4]. However, the accuracy of such intermodal hematoma measurement is not known.

In the hyperacute state, the freshly extravasated blood has similar density as the intravascular blood (30–60 Hounsfield Units, HU), which will soon increase to 80 HU as coagulation proceeds. The high radiodensity allows for an excellent detection rate of intracranial blood with NCCT [5]. CTA has become an integral part of the diagnostic workup because of its high sensitivity for vascular pathologies. In addition, the CTA spot sign, caused by an active extravasation of contrast agent, has been shown to be an indicator of hematoma expansion [6]. CTA is often followed by postcontrast CT to address the presence of underlying neoplastic disease [2].

MRI has been described as accurate as CT in diagnosing the presence of ICH [5]. Furthermore, MRI is the imaging modality of choice to identify CAA or an underlying tumor [7]. However, the MRI appearance of ICH varies in dependence of applied technique, field strength, and the magnetic properties of the hematoma, which change over time [8].

In this study, we retrospectively assessed the volumetric accuracy of different modalities (MRI, CTA, postcontrast CT) to measure hematoma size, in comparison to non-contrast CT in the first 6 days after ICH.

## Methods

### Study population

The data that support the findings of this study are available from the corresponding author upon reasonable request.

We reviewed clinical data collected as part of the prospectively maintained stroke database of our institution (Charité Universitätsmedizin Berlin). It was searched for all patients with the diagnosis of ICH (time span January 2018 and July 2019). Patients > 18 years were included if they had a diagnosis of spontaneous intracerebral hemorrhage (supra- or infratentorial) and had received initial NCCT, followed by CTA, postcontrast CT or cranial MRI within the following 6 days, when ICH is still in its subacute stage [9]. To allow adequate comparison of follow-up imaging to the initial NCCT, a third cranial image was required to exclude patients who experienced interim hematoma growth. Patients referred to our institution from external hospitals were included if initial NCCT was available.

Diagnosis of ICH was established by admission NCCT. On MRI hemorrhage was identified as a characteristic area of hyperintensity or signal loss, respectively, depending on the applied sequence.

The population included patients with anticoagulant treatment, but excluded patients with head trauma, brain tumor, vascular malformation, primary intraventricular hemorrhage, or secondary ICH from hemorrhagic transformation of ischemic infarction. Patients, subjected to surgical procedures (e.g. hematoma evacuation or decompressive craniectomy) were also excluded.

The retrospective study was approved by the responsible ethics committee (Ethik-Komission der Charité Berlin; identification number EA4/011/20). Informed consent of individual patients was waived for this retrospective study with anonymized data according to pertinent institutional guidelines. The study was conducted in accordance with the declaration of Helsinki.

### Image acquisitions

CT scans at the Charité University Hospital were performed on a 80 or 320 slice scanner (Toshiba Aquilion Prime) with the following imaging parameters: NCCT and postcontrast CT with incremental acquisition at 120 kV, 280 mA, 1.0 mm slice reconstruction; postcontrast CT images were acquired 3 min after contrast agent application; CTA: dose-modulated (100–450 mA) spiral CT acquisition at 100–120 kV, 0.5-mm collimation, 0.8 pitch; H20f soft kernel reconstruction, with 1.0 mm standard slices and 5 mm maximum intensity projection (MIP) images with 1 mm increment. 60 mL highly iodinated contrast medium and 30 mL NaCl flush at 4 mL/s; scan triggered by bolus tracking at within the ascending aorta with a 6 s delay.

MR scanners used in this study were newest generation 1.5-T and 3 T (Siemens Medical Solutions, Erlangen, Germany) respectively. A standard head coil was used. The MRI protocol included T2*-Gradient Echo (slice thickness = 2–3 mm), Diffusion weighted imaging (DWI, slice thickness = 2–3 mm), Fluid attenuated Inversion Recovery Sequences (FLAIR, slice thickness = 2–3 mm) and T1w Spin-Echo (slice thickness = 2–3 mm) pre and post gadolinium intravenous administration.

### Image analysis

Two raters, experienced in stroke imaging (F.S., J.K.) independently reviewed images in a random order, blinded to all demographic and outcome data. Readers were not involved in the clinical assessment or care of the enrolled patients. Images were re-randomized and presented again to one rater (F.S.) one month later for a second reading. First, the location of the hematoma and presence of intraventricular hemorrhage were assessed and documented by reviewing admission CT images. Also the hemorrhage locations were classified as deep (basal ganglia and thalamic), lobar, brain stem, and cerebellum on admission CT images. Thereafter, regions of interest (ROIs) were delineated on axial slices of images in the different imaging modalities (admission and follow-up) using a semi-automated segmentation tool provided in the Visage image viewer (version 7.1.10). On CT images measurements were completed between 20 and 80 Hounsfield units (HU) to exclude voxels that likely belong to cerebrospinal fluid or calcification. Discrepancies were settled by joint discussion of the 2 readers.

### Statistical analysis

Clinical data from our stroke database and results from manual segmentation were statistically analyzed as following: For baseline data, mean and standard deviations (SD) were used for normally distributed, and median and range for non-normally distributed data, respectively. To test for normality the D’Agostino-Pearson test was used. Categorical variables are reported as counts and percentages. *P* values less than 0.05 were considered significant. Intrarater and interrater reliability were measured with intraclass correlation coefficients (ICC) using the statistical software package SPSS version 25® (IBM Corporation, Armonk NY). ICC was interpreted as following: Moderate agreement (0.41–0.60), substantial agreement (0.61–0.80), and almost perfect (excellent) (0.81–1.00) [10]. GraphPad Prism 7 version 7.00 was used for Bland–Altman plots to determine the volumetric accuracy of the above mentioned imaging modalities in comparison to initial NCCT.

## Results

From January 2018 to June 2019, 286 patients were identified in our database with the diagnosis of primary acute ICH. Of those, 28 consecutive patients were included, who received initial NCCT as well as at least two follow-up imaging studies, and did not show interim hematoma enlargement. Patients had a median age of 76.5 (66.5–80.5), 15 (53.6%) were female.

Of the 28 patients with initial NCCT 20 received CTA as a follow up, 10 postcontrast CT, and 14 MRI. Duration from initial NCCT to first follow-up imaging was a median of 16 h (IQR 1–28 h); to second follow-up imaging a median of 59 h (IQR 23–98 h). Further baseline demographics and clinical characteristics are shown in Table 1.

**Table 1:** Comparison of baseline demographic, clinical and radiological characteristics in patients with acute intracerebral hemorrhage (ICH)

Baseline characteristics	Patients with acute ICH (n = 28)
*Clinical parameters*	
Age [years], median (IQR)	76.5 (66.5–80.5)
Female, n (%)	15 (53.6)
Hypertension, n (%)	20 (71.4)
Diabetes mellitus, n (%)	3 (10.7)
GCS score, median (IQR)	15 (13–15)
Anticoagulation treatment, n (%)	4 (14.3)
Antiplatelet treatment, n (%)	8 (28.6)
NIHHS admission, median (IQR)	5 (2–11)
NIHSS discharge, median (IQR)	1 (0–8)
mRS discharge, median (IQR)	4 (1–4)
*CT parameters*	
Bleeding location, n (%)	
Lobar	15 (53.6)
Basal Ganglia	6 (21.4)
Thalamus	4 (14.3)
Brainstem and Pons	0 (0.0)
Cerebellar	3 (10.7)
Intraventricular hemorrhage, n (%)	8 (28.6)
Midlineshift [mm], median (IQR)	0.0 (0.0–3.0)

*GCS* Glasgow Coma Scale, *mRS* modified Rankin Scale, *NIHSS* National Institutes of Health Stroke Scale

ICH was deep hemispheric in 10 (35.7%), lobar in 15 (53.6%), and infratentorial in 3 subjects (10.7%). Mean hematoma volume at admission was 18.79 ± 19.86 cc.

All interrater and intrarater ICCs were excellent (1; IQR 0.98–1.00) for both CT and MRI scans.

**Fig. 1:**
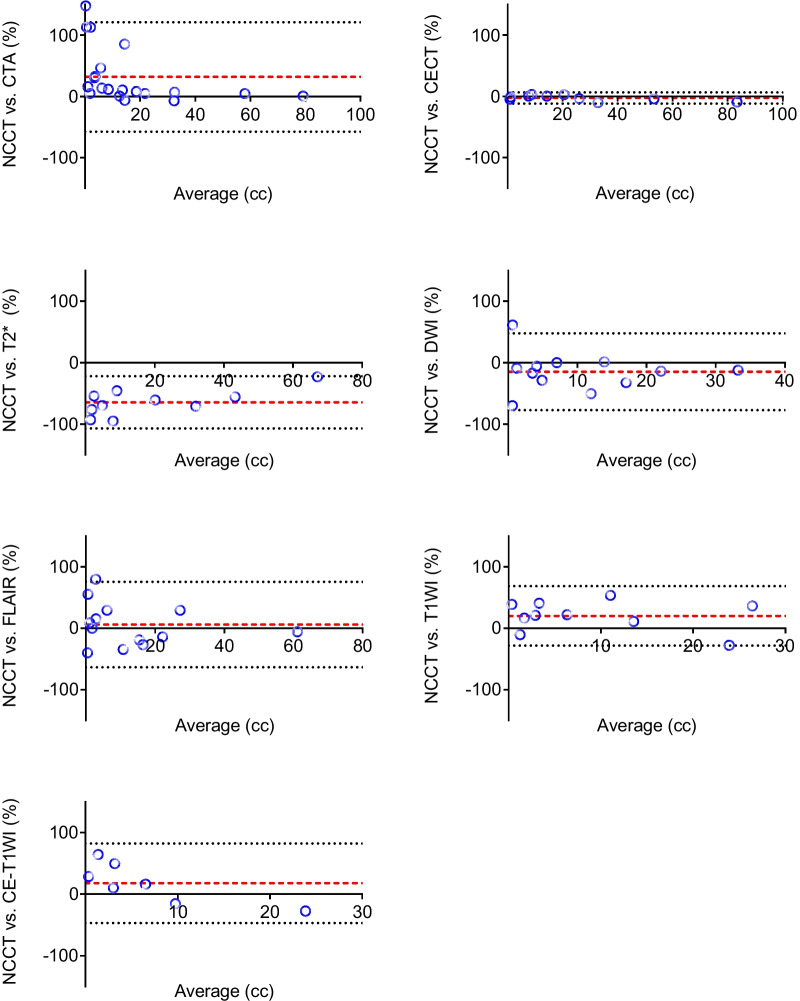
Bland–Altman plots of non-contrast Computed Tomography (NCCT) versus CT-Angiography (CTA) and postcontrast CT (CECT; upper row) and NCCT versus different MRI sequences (second and third row). T2* indicates T2*-Gradient Echo, DWI indicates Diffusion weighted imaging, FLAIR indicates Fluid attenuated Inversion Recovery Sequences, T1WI and CE-T1WI indicate T1w Spin-Echo pre and post gadolinium intravenous administration respectively. Dashed red lines represent the bias (mean of the difference between measurements). Dotted gray lines represent the limits of agreement (mean ± 1.96 SD)

**Fig. 2:**
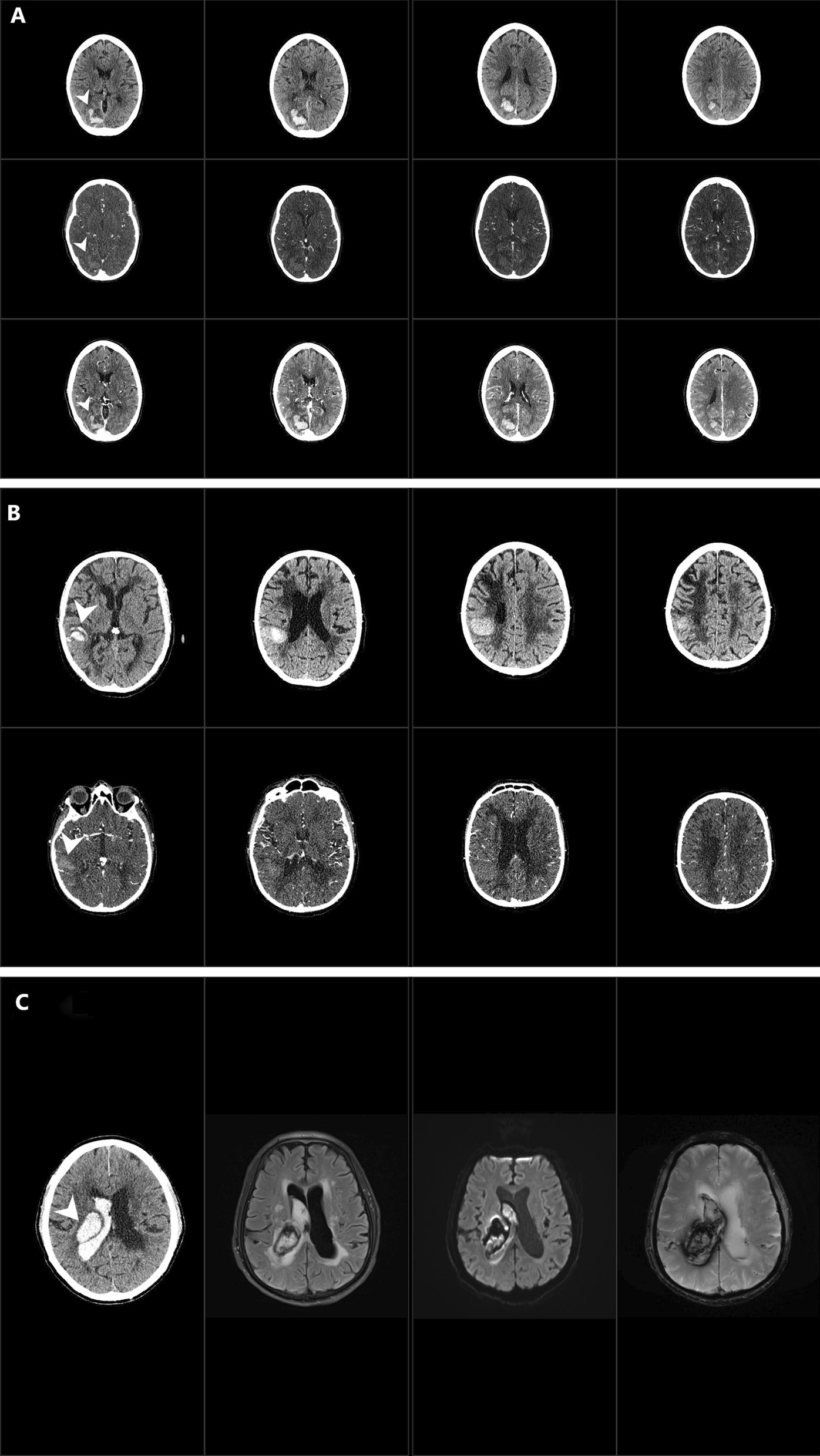
**a** Comparison of the same patient with ICH in the right occipital lobe (arrowhead) using different imaging modalities. The borders of the hematoma can be seen with clear contrast on admission NCCT (upper row) and follow-up postcontrast CT (lower row). On CTA (middle row) borders of the hematoma are difficult to distinguish from surrounding parenchyma. **b** In this example of a patient with right temporoparietal ICH (arrowhead), hematoma appears to be smaller on follow-up CTA (lower row) compared to admission NCCT (upper row). **c** Right thalamic ICH on NCCT, FLAIR, DWI and T2* (from left to right). On MRI sequences hemorrhage are more heterogeneous and borders are not as easily to distinguish from the surrounding tissue. Hemorrhage appears to be bigger on T2*

In comparison to the initial NCCT, volumetric measurements were most accurate in patients who received postcontrast CT (bias of − 2.47%, SD 4.67: n = 10), while CTA often underestimated hemorrhage volumes, especially in bleeds < 20 cc (bias of 31.91%, SD 45.54; n = 20; Bland–Altman plots in Figs. 1, 2a, b; Additional file 1: Table S1).

In MRI sequences ICH volumes were generally overestimated in T2* (bias of − 64.37%, SD 21.65; n = 10). T1WI (bias of 20.23%, SD 24.65; n = 10) and contrast enhanced T1WI (bias of 17.96%, SD 32.9; n = 7) both showed a trend to measure hematoma volumes smaller compared to NCCT. The most accurate MRI sequences in terms of comparability to the initial NCCT were DWI and FLAIR, which showed higher relative deviations in both directions however (bias of-14.6%, SD 31.93; n = 12; bias of 6.05%, SD 35.45; n = 13 respectively; Figs. 1, 2c; Additional file 1: Table S1).

## Discussion

In this retrospective study we investigated the accuracy of volumetric measurements of ICH volumes in different imaging modalities. To our knowledge, this is the first study analyzing the accuracy of volumetric measurements of ICH in NCCT in comparison to CTA, or postcontrast CT. We found an excellent correlation of hemorrhage volume measured on postcontrast CT in comparison to admission NCCT, while hematoma size on CTA was underestimated in some cases. In MRI sequences (DWI, T2*, FLAIR, T1WI with and without CE) hemorrhage size was measured with a substantial bias.

While NCCT has been the gold standard for the diagnosis and for follow-up of ICH for many years, MRI has gained utility in the acute setting, due to its excellent detection rate for ischemic and hemorrhagic stroke. As in a previous study from 1999, T2*-MRI clearly overestimated hematoma size [11]. While that study concluded that DWI and FLAIR were the most accurate MRI techniques, with a good correspondence to NCCT, we also found that DWI and FLAIR were the least inaccurate MRI-sequences, but tended to over- and underestimate, respectively, smaller hematoma volumes < 20 cc. That readers were blinded to initial NCCT results and clinical data in our study (which was not explicitly mentioned in that previous study), the use of current generation MRI and CT hardware, and the fact that we explicitly excluded patients with interim hematoma growth might have contributed to this difference.

The main finding is that postcontrast CT is excellently suited to compare hematoma size with NCCT. This finding could have direct clinical implications, proposing postcontrast CT in patients with high-risk of clinical worsening as the preferred tool (a) to check for further hematoma expansion, (b) to assess venous pooling as a surrogate for active bleeding, and (c) to identify malignancies as underlying pathology [12]. A protocol using a slightly delayed postcontrast CT after initial NCCT would also be a suitable tool in clinical trials targeting hematoma expansion, saving costs and sparing patients substantial radiation dose for an additional NCCT-scan, which would otherwise be the most obvious alternative for precise assessment of hematoma expansion. By contrast, radiologists should be careful to diagnose hematoma progression or regression on CTA, if no additional confirmatory imaging modality is available.

Several strengths of the present study contribute to the robustness of our findings. First, newest generation scanners were used in our study, and second, all images were examined by experienced radiologists blinded to initial NCCT and relevant clinical data. Limitations are the retrospective design, and the moderate sample size. While similar case numbers can be found for comparable studies in the literature we understand our work as a pilot study. Careful interpretation is therefore warranted and prospective validation of the results in a larger patient cohort will be needed in the future [11, 13, 14]. While our dataset comprised both small and large hemorrhages, clinically severe cases of ICH might be underrepresented in our dataset (GCS 15 (13–15; Additional file 2: Table S2)). Furthermore we did not include data on intraventricular hemorrhage, which we plan to investigate in future work.

## Conclusions

Postcontrast CT offers excellent interrater and intrarater reliabilities to measure hematoma size in follow-up imaging after acute ICH and offers a rationale for a standardized follow-up imaging protocol. Hematomas on CTA should not be compared with initial NCCT since it regularly underestimates hematoma volumes. While MRI has excellent sensitivity for the qualitative detection of ICH, quantitative measurement of hematoma size may not be sufficiently accurate for a precise comparison with NCCT acquired on admission.

## Supplementary Information

**Additional file 1: Table S1.** Absolute ICH volumes in non-contrast Computed Tomography (NCCT; left side) and follow-up imaging modality (right side).

**Additional file 2: Table S2.** Comparison of baseline demographic, clinical and radiological characteristics in patients with acute intracerebral hemorrhage (ICH; study cohort vs. comparison group). EVD indicates extraventricular drainage; GCS, Glasgow Coma Scale; mRS modified Rankin Scale; NIHSS National Institutes of Health Stroke Scale.

## Data Availability

The data that support the findings of this study are available from the corresponding author upon reasonable request.

## Abbreviations

MRI: Magnetic resonance imaging
CTA: Computed tomography angiography
CT: Computed tomography
ICH: Intracerebral hemorrhage
ICC: Intraclass correlation coefficient
IQR: Interquartile range
NCCT: Non-contrast computed tomography
CAA: Cerebral amyloid angiopathy
kV: Kilovolt
mA: Milliampere
MIP: Maximum intensity projection
NaCl: Sodium chloride
T: Tesla
DWI: Diffusion weighted imaging
FLAIR: Fluid attenuated inversion recovery
T1WI: T1 weighted image
ROI: Region of interest
HU: Hounsfield units
SD: Standard deviation
CE: Contrast Enhanced
GCS: Glasgow coma score
