# Supplementary material for: Volumetric accuracy of different imaging modalities in acute intracerebral hemorrhage
Source: BMC Med Imaging. 2022 Jan 15;22:9. doi: 10.1186/s12880-022-00735-3 (PMC8760700; doi:10.1186/s12880-022-00735-3)
Supplement: Supplementary file 1 — Additional file 1: Table S1. Absolute ICH volumes in non-contrast Computed Tomography (NCCT; left side) and follow-up imaging modality (right side). [file 12880_2022_735_MOESM1_ESM.docx]

**Additional file 1: Table S1**

| **NCCT [cc]. median (IQR)** | **Follow-up imaging [cc]. median (IQR)** |
| --- | --- |
| 10.79 (3.395-21.88) | CTA: 8.135 (2.038-20.38); (n=20) |
| 17.55 (6.01-36.45) | CECT: 17.2 (5.975-39.45); (n=10) |
| 5.62 (1.668-23.25) | T2*: 11.5 (3.05-46.23); (n=10) |
| 5.62 (1.685-14.23) | DWI: 6.32 (1.915-18.6); (n=12) |
| 7.04 (1.505-17.45) | FLAIR: 5.24 (1.395-20.95); (n=13) |
| 5.51 (1.668-15.88) | T1WI: 4.135 (1.49-15); (n=10) |
| 3.98 (1.83-8.97) | CE-T1WI: 2.89 (0.94-10.5); (n =7) |

*Legend*: Absolute ICH volumes in non-contrast Computed Tomography (NCCT; left side) and follow-up imaging modality (right side).
