# Supplementary material for: Volumetric accuracy of different imaging modalities in acute intracerebral hemorrhage
Source: BMC Med Imaging. 2022 Jan 15;22:9. doi: 10.1186/s12880-022-00735-3 (PMC8760700; doi:10.1186/s12880-022-00735-3)
Supplement: Supplementary file 2 — Additional file 2: Table S2. Comparison of baseline demographic, clinical and radiological characteristics in patients with acute intracerebral hemorrhage (ICH; study cohort vs. comparison group). EVD indicates extraventricular drainage; GCS, Glasgow Coma Scale; mRS modified Rankin Scale; NIHSS National Institutes of Health Stroke Scale. [file 12880_2022_735_MOESM2_ESM.docx]

**Additional file 2: Table S2**

| Baseline characteristics | | Study cohort (n = 28) | Comparison group (n = 200) | *P*-Value |
| --- | --- | --- | --- | --- |
| *Clinical parameters* | | | |  |
| Age [years], median (IQR) | 76.5 (66.5-80.5) | | 72 (58.75-80.0) | 0.34 |
| Female, n (%) | 15 (53.6) | | 113 (65.5) | 0.31 |
| Hypertension, n (%) | 20 (71.4) | | 61 (81.5) | 0.84 |
| Diabetes mellitus, n (%) | 3 (10.7) | | 33 (16.5) | 0.58 |
| GCS score, median (IQR) | 15 (13-15) | | 12 (6.5-14) | 0.001 |
| Anticoagulation Treatment, n (%) | 4 (14.3) | | 57 (28.5) | 0.16 |
| Antiplatelet Treatment, n (%) | 8 (28.6) | | 38 (19) | 0.16 |
| NIHHS admission, median (IQR) | 5 (2-11) | | 10 (4-13) | 0.26 |
| NIHSS discharge, median (IQR)  mRS discharge, median (IQR) | 1 (0-8)  4 (1-4) | | 5 (1-12)  5 (4-5.5) | 0.15  0.007 |
| *CT parameters* | | | |  |
| Bleeding location,  n (%)   - Lobar - Basal Ganglia - Thalamus - Brainstem/Pons - Cerebellar | 15 (53.6)  6 (21.4)  4 (14.3)  0 (0.0)  3 (10.7) | | 86 (43)  68 (34)  14 (7)  11 (5.5)  18 (9) | 0.32  0.17  0.012  0.35  0.21 |
| Intraventricular hemorrhage, n (%) | 8 (28.6) | | 87 (43.5) | 0.44 |
| Midlineshift [mm], median (IQR) | 0.0 (0.0-3.0) | | 2 (0.0-6.0) | 0.018 |

*Legend*: Comparison of baseline demographic, clinical and radiological characteristics in patients with acute intracerebral hemorrhage (ICH; study cohort vs. comparison group). EVD indicates extraventricular drainage; GCS, Glasgow Coma Scale; mRS modified Rankin Scale; NIHSS National Institutes of Health Stroke Scale.
